# Investigating current and temporal variation in municipal youth smoking rates in the Netherlands: A multivariable regression analysis

**DOI:** 10.18332/tid/209127

**Published:** 2025-10-09

**Authors:** Sophie J. A. Jooren, Jeroen Bommelé, Ben Wijnen, Wouter den Hollander, Jessica Baars, Maria W. J. Jansen, Marc C. Willemsen

**Affiliations:** 1 Department of Health Promotion, School of Public Health and Primary Care, Maastricht University, Maastricht, The Netherlands; 2 Trimbos Institute, The Netherlands Expertise Centre for Tobacco Control, Utrecht, The Netherlands; 3 Centre for Economic Evaluations, Trimbos Institute, Netherlands Institute of Mental Health and Addiction, Utrecht, The Netherlands; 4 Department of Epidemiology, Trimbos Institute, Netherlands Institute of Mental Health and Addiction, Utrecht, The Netherlands; 5 Health Funds for a Smokefree Netherlands, Utrecht, The Netherlands; 6 Department of Health Services Research, School of Public Health and Primary Care, Maastricht University, Maastricht, The Netherlands

**Keywords:** smoking municipalities, youth, smoking differences

## Abstract

**INTRODUCTION:**

Variations in smoking prevalence rates exist across different regions. While most research tends to study demographic, cultural or social determinants of smoking, few studies have looked into whether municipal-level variables are associated with smoking rates, especially among the younger population. We examined which municipal-level factors explain differences in youth smoking rates in the Netherlands.

**METHODS:**

We conducted linear regression analyses to examine municipal-level smoking behavior among the population aged 12–16 years. Main outcomes were the proportion of youth within municipalities that had ever smoked or weekly smoked in 2021, and trend differences in ever smoking and weekly smoking between 2015 and 2021. Independent variables were population density, level of urban density, proportion of people with a migration background, proportion of elderly residents, and proportion of adults with a low level of education. We examined municipalities' involvement in the Smoke-free Generation campaign, their tobacco control policies, the number of smoke-free outdoor areas implemented, and their self-assessed tobacco control scores.

**RESULTS:**

Dutch municipalities show great variation in smoking prevalence (2021: ever smoking 3.0–21.8% and weekly smoking 1.4–11.1%). Smoking has decreased in almost all municipalities in recent years; 108 of the 113 municipalities had a decrease in ever smoking, while 89 of the 100 municipalities showed a decrease in weekly smoking prevalence. Municipalities with a higher proportion of individuals with a migration background had lower ever (β= -0.08, p=0.021) and weekly (β= -0.06, p=0.013) youth smoking prevalence rates in 2021.

**CONCLUSIONS:**

Considering that municipality-specific variables were not associated with levels of youth smoking prevalence, the question remains why some municipalities have much higher smoking rates than others, after having been exposed to the same national level tobacco control policy measures.

## INTRODUCTION

Across the globe, countries are actively addressing and monitoring the impact of tobacco use and exposure to tobacco smoke among adult and youth populations. While global guidelines within the Framework Convention on Tobacco Control (FCTC) for implementing tobacco control measures exist, substantial variation in smoking persists among countries^1^.

In Europe, the Tobacco Control Scale ranks European countries according to the extent of which tobacco control measures have been implemented by each respective country^2^. Ireland, for example, has the most comprehensively implemented tobacco control measures (current smoking prevalence: 17%), while Bosnia-Herzegovina has the lowest score on the Tobacco Control scale (current smoking prevalence: 40%)^3,4^.

Also, within countries, notable variations in smoking prevalence rates exist across regions and municipalities. In Belgium, for example, the smoking prevalence rates among municipalities range between 11% to 27%^5^. Among municipalities of Austria, smoking rates range between 14% and 31%^6^. In the Netherlands, smoking rates among municipalities range from 8% to 24%^7^. Within these variations, a specific difference has been observed between urban and non-urban regions in countries. Idris et al.^8^ showed that smoking prevalence rates are directly related to urban density, with the highest prevalence rate in the most urbanized areas. Tomintz et al.^6^ and Bommelé et al.^9^ also reported higher smoking rates in Dutch regions with a higher proportion of urban areas.

Several explanations have been proposed for the differences within countries. Some researchers have pointed to region-specific demographics (such as education level), as cultural or social factors explanations. For example, Indris et al.^8^ argued that urban differences in smoking prevalence could not solely be explained by education level, income, and occupation of residents in certain areas, and highlight factors like migration background and urban environment’s influence. Furthermore, urban areas may have more permissive smoking norms, promoting smoking initiation and hindering smoking cessation. Another study found that smoking rates have decreased in rural municipalities^6^. This is likely due to an ageing population, as younger people move to cities, birth rates decline, and older people are less likely to smoke. Finally, Mlinarić et al.^10^ compared smoking bans across seven cities, finding differences due to varying municipality-specific context factors such as collaboration levels between local or regional NGOs, enforcement strategies by environmental health departments, police or enforcement officers, consumers’ and food safety authorities, and existing legislation.

In trying to explain differences between and within countries, most studies compared smoking rates in the adult population. No analyses of younger populations have been carried out in any of these studies. However, when trying to control smoking rates, young people, especially those below 18 years of age, are an important target group^11^. Notable differences exist in smoking prevalence rates among young people across countries. Within the European Union, for example, daily smoking rates among people aged 15–19 years differed in 2019 from 7.3% in Luxembourg to 28.6% in Hungary. Also, within countries, there are notable differences among regions^12^. However, no studies have investigated youth smoking differences between municipalities within a country or to what extent municipality-specific variables are associated with such differences in youth smoking rates. In this study, we investigate which municipal-level variables (both demographic and policy-related) explain differences in municipal youth smoking rates in the Netherlands. We do this for 2021 smoking rates and for smoking trends between 2015 and 2021.

## METHODS

### Study design, sample characteristics and sample size

We used three existing datasets and merged these into one dataset with municipality as the unit of analysis. We combined data from the Youth Health Monitor^13-15^, data from the Location Monitor, and data from Statistics Netherlands (see below). The Youth Health Monitor included the main outcome: municipal youth smoking rates (‘ever smoking’ and ‘weekly smoking’). The Location Monitor was used for data on the implementation of local tobacco control policies. Statistics Netherlands provided data on population characteristics of municipalities. Table 1 lists each included variable and its source. Below, we describe each of the data sources in detail.

**Table 1:** Sample characteristics of municipalities by outcome (rates of ever smoking and weekly smoking among the population aged 12–16 years), Netherlands, 2021

*Characteristics*	*Ever smoking rates 2021 (outcome)*				*Weekly smoking rates 2021 (outcome)*			
	*N*	*Mean (range)*	*Median*	*SD*	*N*	*Mean (range)*	*Median*	*SD*
Average smoking rates (2021 minus 2015) within municipalities of population aged 12–16 years	301	9.8 (3.0–20.3)	9.6	3.4	265	4.7 (1.38–11.14)	4.4	2.08
Inhabitants per km^2^	301	1071.6 (71.0–6650.0)	590	1190.1	265	1028 (1136.7–71.0)	587	1136.7
Urban density	301	2.8 (1.0–5.0)	3.0	-	265	2.9 (1.0–5.0)	3.0	
Mean standardized income	301	36.0 (29.1–66.8)	35.0	5.3	265	35.9 (28.2–66.8)	35.0	5.1
Proportion of residents with a migration background (%)	301	18.0 (4.3–56.2)	15.4	9.7	265	17.9 (4.3–56.2)	15.6	9.4
Proportion of elderly residents (%)	301	21.6 (9.8–32.9)	21.6	3.5	265	21.6 (9.8–32.9)	21.6	3.4
Proportion of adults with a low level of education (%)	301	22.5 (14.4–29.3)	22.7	2.9	265	22.7 (14.4–32.9)	22.6	3.1
Being actively involved in the Smoke-free Generation movement (%)	225	87 (0–100)	100	34	198	86 (0–100)	100	34
Smoking being incorporated in a local policy (%)	179	69 (0–100)	100	47	157	69 (0–100)	100	46
Number of smoke-free location types	192	5.1 (0.0–13.0)	5.0	3.2	167	5.2 (0.0–13.0)	5.0	3.1
Tobacco control implementation grade	216	6.6 (3.0–10.0)	7.0	1.4	189	6.6 (3.0–10.0)	7.0	1.4

### Youth health monitor – dependent variable

The Youth Health Monitor provides insight into the health, well-being and substance use of secondary school students (12–16 years)^13-15^. The survey is conducted once every four years by all regional public health services in the Netherlands. The data collection is coordinated by GGD GHOR Netherlands (the Association for Public Health and Safety in the Netherlands) and the National Institute for Health and Environment (RIVM). In 2015, 97000 students at 377 schools participated. In 2021, 167000 students at 759 schools participated.

We were interested in two main outcome variables: smoking prevalence in 2021 for each municipality and the difference in smoking prevalence rates between 2015 and 2021 per municipality. These variables had been aggregated to the municipality level from original individual level data from the Youth Health Monitor. We were interested in ever smoking and weekly smoking. The following questions were asked in 2015 and 2021: ‘Have you ever smoked? By this we mean cigarettes and rolling tobacco, not electronic cigarettes’. Response options were: ‘Yes, a whole cigarette or more’, ‘Yes, just a few puffs’, and ‘No’. Follow-up question for those who answered ‘yes’ was: ‘How often do you smoke now?’. Response options: ‘Every day’, ‘At least once a week, but not every day’, ‘Less than once a week’, and ‘I don’t smoke’. For the analyses, we used ever and weekly smoking. The first was ever smoking a cigarette. This had been measured by asking respondents whether they have ever smoked at least one whole cigarette. The other outcome measure was weekly smoking. This had been measured by asking whether they smoke at least once a week, but not every day.

Participants had provided their postal code at the start of the questionnaire. This information was used to identify respondents’ municipality. For each municipality, the smoking prevalence had been calculated based on the average of all participants within that municipality, resulting in a percentage of young people who had ever smoked or smoked weekly. These local municipal smoking rates were used in the analyses as outcome variable.

The municipal-level data were weighted by municipality, gender, grade level, and type of education. Finally, data were available for 122 of 393 municipalities in 2015, and 301 of 352 in 2021. Municipalities were missing if: 1) only a small number of participants had completed the questionnaire within a municipality, resulting in the exclusion of that municipality to protect participants’ identification; and 2) there had been no participants in a municipality that participated in the survey. Between 2015 and 2021, some municipalities merged into larger municipalities. To account for this, data were recalculated according to the geographical boundaries of municipalities present in 2021, i.e. when the Netherlands included 352 municipalities. Additional information on the data can be found on the Health Monitor website^16^. Trend differences in smoking rates were calculated by subtracting the smoking prevalence in 2021 from that in 2015.

### Location monitor (policy-related variables)

Data on municipal tobacco control policies were provided by Health Funds for Smoke Free, which commissions the Location Monitor yearly since 2020. Data collection had been conducted by I&O Research^17,18^. I&O Research approached specific people within municipalities (policy officers on health, exercise, sports, etc.) or e-mailed a more general municipal e-mail address. A total of 256 municipalities completed the questionnaire in 2021 (response rate: 73%). We used the following four items from the Location Monitor as independent variables:

1. Whether a municipality was actively supporting the Smoke-free Generation movement was measured with the item: ‘Is your municipality active in the field of the Smoke-free Generation?’. Answer options were ‘Yes’, ‘Yes, but not under the name of the Smoke-free Generation’, ‘No’, and ‘I don’t know’. We recoded this variable into a binary variable. The answers ‘Yes’ and ‘Yes, but not under the name of the Smoke-free Generation’ were coded as 1 and ‘No’ was coded as 0. ‘I don’t know’ was coded as missing. The Smoke-free Generation is a national movement in the Netherlands aimed at ensuring that all children born from 2017 onward grow up in a completely smoke-free environment^19^. Initiated by a collaboration between the Dutch Heart Foundation, the Dutch Cancer Society, and the Lung Foundation Netherlands, the campaign seeks to de-normalize smoking and reduce exposure to tobacco smoke in public spaces.
2. Having tobacco control incorporated in a local policy was measured with the item: ‘Has this [Smokefree Generation policy] been included in formal policy documents?’. Answer options: ‘Yes’, ‘No’, ‘I don’t know’. We recoded this variable into a binary variable. ‘Yes’ was coded as 1 and ‘No’ was coded as 0. ‘I don’t know’ was coded as missing.
3. Number of smoke-free location types within the municipality was measured with the item: ‘At which locations does your municipality play (or played) a role in creating smoke-free outdoor spaces?’. For each location type municipalities indicated if they had created a smoke-free policy or helped others create one. We calculated a composite score of all smoke-free outdoor location types in which the municipality was involved in (range: 0–14). Possible location types were: association playground(s), municipal sports field, municipal playgrounds and play areas, outdoor sports location, outdoor swimming pool, petting zoo, scout group(s) with a scouting area, childcare locations (site), school grounds, amusement park and/or zoo, area of a healthcare institution, public area for the entrance of locations, public outdoor space (e.g. a street or square), and the entrance of town halls.
4. Striving for a Smoke-Free Generation within the municipality was measured with the item: ‘To what extent do you think your municipality strives for a Smoke-Free Generation?’. Respondents rated the extent on a 0–10 Likert scale, where higher scores indicate that a municipality believes they contributed strongly to the Smoke-Free Generation movement.

### Municipal-level demographic variables

Demographic characteristics of municipalities were obtained from Statistics Netherlands^20,21^. We obtained the following data for each of the 352 municipalities: 1) Number of residents per km^2^, 2) average standardized income (average standardized income (× 1000 euros) (mean), 3) proportion of inhabitants with a migration background, 4) urban density level of the municipality (coded using levels from 1 to 5 with 1 being not urban, 2 slightly urban, 3 moderately urban, 4 strongly urban, and 5 very highly urban), 5) percentage of elderly people within the municipality (i.e. people older than 64 years), and 6) the percentage of residents with a low level of education. Except for education level (only measured in 2019), all demographic characteristics were available for 2021 according to the municipality classification of 2021. Urban density levels (addresses/km^2^) were defined as follows: not urban <500, slightly urban 500–1000, moderately urban 1000–1500, strongly urban 1500–2500, and very highly urban ≥2500. We chose these characteristics based on availability and former literature, as outline in the introduction.

### Analyses

Four linear multivariable regression analyses were performed, with each analysis consisting of two models with different sets of independent variables. In Model 1, we included the municipal-level demographic variables (inhabitants per km^2^, migration background, level of urban density, proportion of elderly residents, and proportion of adults with a low level of education). In Model 2, we added four policy-related variables: being actively involved in the Smoke-free Generation movement, smoking being incorporated in a local policy, number of smoke-free location types, and self-reported tobacco control grade.

*Ever smoking*

The first set of multivariable regression analyses included municipal ever smoking rates among youth in 2021 from the Youth Health Monitor as the dependent variable for both models. Independent factors for the Model 1 included only the municipal-level demographic variables. In Model 2, we added the four policy variables.

*Weekly smoking*

The second set of multivariable regression analyses included municipal weekly smoking rates among youth in 2021 from the Youth Health Monitor as the dependent factor for both models. Included independent variables for the Model 1 and the Model 2 were equal to the first analyses.

*Changes in ever and weekly smoking over time*

For the third and fourth set of multivariable regression analyses, differences were calculated between the ever smoking rates and weekly smoking rates in 2015 and 2021. For both sets, differences in these rates (2021 minus 2025) were included as the dependent variable for both models. Again, included independent variables for the Model 1 and Model 2 were equal to the first analyses.

All analyses were two-tailed with a significance level of p<0.05. Given that multiple datasets with varying numbers of municipalities were combined, the sample size differed across models. None of the models included all 352 municipalities present in the Netherlands in 2021. We obtained data on weekly smoking for 265 municipalities, while we had data on ever smoking for 301 municipalities. To examine if bias was introduced, an additional logistic analysis was performed for each of the two models to determine whether the included municipalities significantly differed from the excluded municipalities. These additional logistic analyses can be found in the Supplementary file Appendix B.

Moreover, given that the *a prior* selected predictors may not contribute to the most optimal model (e.g. due to added ‘noise’ of poorly performing predictors), we performed backward regression for every model to simplify each model by removing variables that do not significantly contribute to explaining the proportion of the variance in de outcome variable (adjusted R^2^). All analyses were performed with R version 4.3.0.

## RESULTS

**Figure 1:**
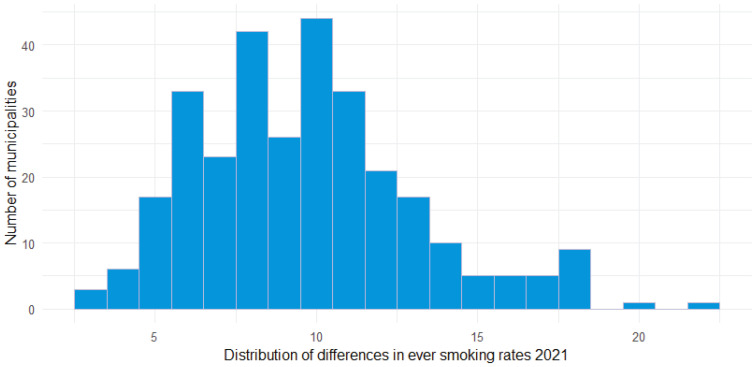
Distribution of differences between municipalities in ever smoking rates among the population aged 12-16 years, Netherlands, 2021

**Figure 2:**
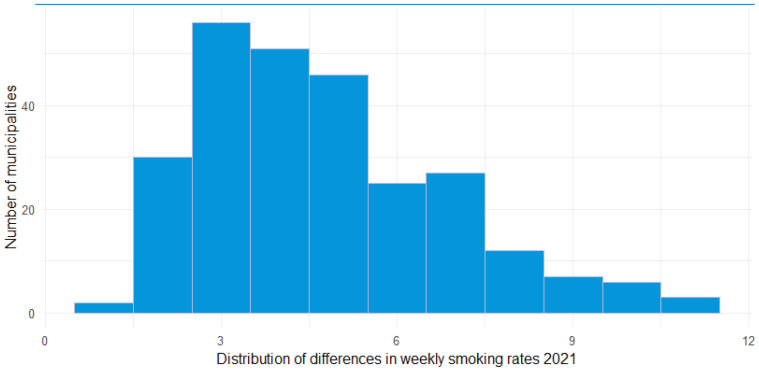
Distribution of differences between municipalities in weekly smoking rates among the population aged 12-16 years, Netherlands, 2021

Tables 1 and 2 describe characteristics of the sample of municipalities included. Tables 3 and 4 show the results from the regression analyses. Figure 1 shows the differences between municipalities for ever smoking rates in 2021. Figure 2 shows the differences between municipalities for weekly smoking rates in 2021.

**Table 2:** Sample characteristics of municipalities by outcome (rates of difference in ever smoking and weekly smoking among the population aged 12–16 years), Netherlands, 2015–2021

*Characteristics*	*Difference in ever smoking 2015–2021*				*Difference in weekly smoking 2015–2021*			
	*N*	*Mean (range)*	*Median*	*SD*	*N*	*Mean (range)*	*Median*	*SD*
Difference ever and weekly smoking (2021 minus 2015) within municipalities of population aged 12–16 years	113	-5.6 (-14.7–3.3)	-5.6	3.6	100	-2.9 (-9.0–3.1)	2.6	2.6
Inhabitants per km^2^	113	1025 (84.0–5573.0)	535	1102	100	1156.7 (127.0–5573.0)	726.0	1256.2
Urban density	113	2.8 (1.0–5.0)	3.0	-	100	2.9 (1.0–5.0)	3.0	-
Mean standardized income	113	36.0 (28.3–66.8)	35.2	4.7	100	36.0 (30.3–66.8)	34.8	5.6
Proportion of residents with a migration background (%)	113	17.6 (4.8–52.9)	14.9	8.4	100	18.5 (5.5–44.6)	15.8	8.7
Proportion of elderly residents (%)	113	22.4 (15.4–32.9)	22.2	3.4	100	22.5 (16.3–32.9)	22.2	3.4
Proportion of adults with a low level of education (%)	113	22.9 (15.2–32.9)	23.0	3.3	100	23.1 (15.3–29.3)	23.1	3.1
Being actively involved in the Smoke-free Generation movement (%)	85	81 (0–100)	100	39	75	79 (0–100)	100	41
Smoking being incorporated in a local policy (%)	64	59 (0–100)	100	50	55	56 (0–100)	100	50
Number of smoke-free location types	68	4.6 (0.0–12.0)	4.0	2.8	58	4.7 (0.0–12.0)	4.0	2.8
Tobacco control implementation grade	82	6.2 (3.0–9.0)	6.0	1.3	72	6.3 (3.0–9.0)	6.0	1.3

**Table 3:** Multivariable regression analysis of ever smoking among the population aged 12–16 years and characteristic of municipalities (Model 1) and characteristics of municipalities and tobacco control variables (Model 2), Netherlands, 2021

*Variables*	*Model 1* *(N=301)*			*Model 2* *(N=179)*		
	*β*	*95% CI*	*p*	*β*	*95% CI*	*p*
Number of inhabitants per km²	0.00	-0.00–0.00	0.870	0.00	-0.00–0.00	0.471
Level of urban density	-0.19	-0.76–0.38	0.520	0.00	-0.71–0.72	0.990
Mean standardized income	-0.02	-0.13–0.08	0.614	0.03	-0.13–0.12	0.573
Proportion of residents with a migration background	-0.08	-0.15 – -0.01	**0.021**	-0.11	-0.20 – -0.02	**0.013**
Proportion of elderly residents	0.08	-0.06–0.22	0.255	0.06	-0.10–0.23	0.449
Proportion of adults with a low level of education	-0.04	-0.22 - 0.13	0.618	-0.09	-0.32–0.14	0.442
Being actively involved in the Smoke-free Generation movement	-	-	-	NA	NA	NA
Smoking being incorporated in a local policy	-	-	-	-0.13	-1.28–1.02	0.827
Number of smoke-free location types	-	-	-	-0.14	-0.32–0.03	0.110
Tobacco control implementation grade	-	-	-	0.37	-0.06–0.81	0.093

Model 1: includes the independent variables inhabitants per km^2^, level of urban density, standard income (mean), migration background, proportion of elderly residents, and education low. Model 2: includes the independent variables inhabitants per km^2^, level of urban density, standard income (mean), migration background, proportion of elderly residents, proportion of adults with a low level of education, being actively involved in the Smoke-free Generation movement, smoking being incorporated in a local policy, number of smoke-free location types, and tobacco control implementation grade.

**Table 4:** Multivariable regression analysis of the association between municipality rate of weekly smoking among the population aged 12–16 years and characteristic of municipalities (Model 1) and characteristics of municipalities and tobacco control variables (Model 2), Netherlands, 2021

*Variables*	*Model 1* *(N=265)*			*Model 2* *(N=157)*		
	*β*	*95% CI*	*p*	*β*	*95% CI*	*p*
Number of inhabitants per km²	0.00	-0.00–0.00	0.775	0.00	-0.00–0.00	0.635
Level of urban density	-0.21	-0.57–0.16	0.267	0.01	-0.44–0.11	0.970
Mean standardized income	0.00	-0.06–0.06	0.996	0.03	-0.04–0.11	0.399
Proportion of residents with a migration background	-0.06	-0.10 – -0.01	**0.013**	-0.08	-0.13 – -0.02	**0.006**
Proportion of elderly residents	0.00	-0.09–0.09	0.995	-0.02	-0.13–0.08	0.682
Proportion of adults with a low level of education	0.03	-0.08–0.15	0.539	0.03	-0.11–0.17	0.663
Being actively involved in the Smoke-free Generation movement	-	-	-	NA	NA	NA
Smoking being incorporated in a local policy	-	-	-	0.06	-0.68–0.80	0.871
Number of smoke-free location types	-	-	-	-0.06	-0.68–0.80	0.337
Tobacco control implementation grade	-	-	-	0.11	-0.17–0.40	0.437

Model 1: demographic level model includes the independent variables inhabitants per km^2^, level of urban density, standard income (mean), migration background, proportion of elderly residents, and proportion of adults with a low level of education. Model 2: includes the independent variables inhabitants per km^2^, level of urban density, standard income (mean), migration background, proportion of elderly residents, proportion of adults with a low level of education, being actively involved in the Smoke-free Generation movement, smoking being incorporated in a local policy, number of smoke-free location types, and tobacco control implementation grade.

### Ever smoking

Table 3 presents multivariable regression analyses for municipal ever smoking rates, both for the Model 1 and Model 2 (Model 1: adjusted R^2^=0.07, F=4.54, p<0.001; Model 2: adjusted R^2^=0.06, F=2.16, p=0.027). In both models, only the proportion of inhabitants with a migration background was found to have a significant association with municipal ever smoking rates (Model 1: β= -0.08, p=0.021; Model 2: β= -0.11, p=0.013). With backward regression for both models, the proportion of inhabitants with a migration background remained as the only significant variable in the model. Thus, municipalities with a higher proportion of people with a migration background had a lower prevalence of youth ever smoking in 2021.

### Weekly smoking

Table 4 presents multivariable regression analyses for municipal weekly smoking rates, both for the Model 1 and Model 2 (Model 1: adjusted R^2^=0.09, F=5.34, p<0.001; Model 2: adjusted R^2^=0.07, F=2.40, p=0.015). In the multivariable regression analysis, proportion of inhabitants with a migration background had a significant negative effect both in the Model 1 (β= -0.06, p=0.013) and Model 2 (β= -0.07, p=0.006). Thus, municipalities with a higher proportion of people with a migration background had a lower prevalence of youth weekly smoking in 2021.

### Changes in ever smoking over time

In 108 municipalities we observed a decrease in smoking: 46 municipalities had a decrease between the 0% and 5%, 48 municipalities had a decrease between 5% and 10%, while 14 municipalities saw a decrease in smoking between 10% and 15%. In contrast, five municipalities had an increase between the 0% and 5%. Supplementary file Figure 1 displays the distribution of the differences in smoking prevalence of municipalities between 2015 and 2021. When explaining these changes over time from the municipality level factors, we found that the Model 1 (adjusted R^2^= -0.04, F=0.23, p=0.967) and Model 2 (adjusted R^2^= -0.04, F=0.23, p=0.967) both had a negative R^2^, meaning that our explanatory model is worse than a model without predictors. Similarly, in the backward regression all variables were removed from the model. Supplementary file Table S1 presents the multivariable regression for ever smoking over time.

### Changes in weekly smoking over time

In all, 89 municipalities had a decrease in smoking prevalence, 74 municipalities had a decrease between 0% and 5%, 15 municipalities had an decrease between 5% and 10%, and 11 municipalities had an increase between 0% and 5%. Supplementary file Figure 2 displays the distribution of the differences in smoking prevalence of municipalities between 2015 and 2021. Model 1 (adjusted R^2^= -0.04, F=0.23, p=0.967) and Model 2 (adjusted R^2^= -0.04, F=0.76, p=0.649) both have a negative R^2^. Consequently, in the backward regression all variables were removed from the model. Supplementary file Table S2 presents the multivariable regression for ever smoking over time.

Table 1 represents the sample characteristics of municipalities represented by outcome variable (ever smoking 2021 and weekly smoking 2021). While data on weekly smoking rates are more limited, the characteristics of the municipalities show little variation. Notable observations in the table include the higher rates of smoking among ever smokers compared to weekly smokers among youth in municipalities. Many municipalities are actively involved in the Smoke-Free Generation initiative, with 69% of municipalities in both samples having formally established their policies. On average, municipalities rate themselves a 6.6 (on a 1–10 point rating scale) regarding how well they are doing in relation to implementing Smoke-Free Generation, tobacco control activities.

Table 2 represents the sample characteristics of municipalities represented by outcome variable (difference in ever smoking, and in weekly smoking, between 2015 and 2021). While data on difference in weekly smoking rates are more limited, the characteristics of the municipalities show little variation. Many municipalities are actively involved in the Smoke-Free Generation initiative, with approximately 60% of municipalities in both samples having formally established their policies. On average, municipalities rate themselves a 6.0 (on a 1–10 point rating scale) regarding how well they are doing in relation to implementing Smoke-Free Generation tobacco control activities.

The additional multivariable logistic regression analysis between the included and excluded municipalities for each model are presented in Supplementary file Tables S3–S6. Overall, we see that municipalities with a strong level of urban density had often a higher chance to participate in the questionnaire: Table S3 (Model 1: β=3.04, p=0.008; Model 2: β=3.87, p=0.048), Table S4 (Model 2: β=2.36, p=0.035), and Table S6 (Model 1: β=1.27, p=0.031). In some cases, a higher population of low level of education (Table S3, β=0.26, p=0.033), and a higher proportion of elderly residents in their municipality (Table S5, β=0.10, p=0.020; β=0.17, p=0.004), Table S6 (β=0.15, p=0.012) had a higher chance of participating in the questionnaire.

## DISCUSSION

We found that in 2021, for the population aged 12– 16 years for both ever smoking and weekly smoking, Dutch municipalities show great variation in smoking prevalence (2021: ever smoking 3.0–21.8% and weekly smoking 1.4–11.1%). This is also the case for the changes in smoking rates over time. While in most municipalities youth smoking rates went down between 2015 and 2021 – with some showing strong reductions – in a few municipalities smoking rates increased. The decrease varied between 0% and 15%. The increase varied between 0 and 5%.

### Associations between youth smoking rates within municipalities and demographic variables of municipalities

We expected to find several associations between youth smoking rates and background variables. Based on the literature, we anticipated, for example, that urbanization and the number of inhabitants per km^2^ would show an effect on smoking. We expected that municipalities with higher levels of urbanization would also have higher smoking prevalence, as seen in former studies^6,8^. Furthermore, the literature suggests that smaller municipalities face more challenges in implementing tobacco control policies than larger ones^22,23^. Therefore, we also expected that the number of inhabitants per km^2^ would show an effect. However, none of these variables showed an association with youth smoking rates in the analyses.

A reason for not finding expected associations could be that predictors used in the analyses might have an effect in real-life, which our data were unable to detect. This might be the case for urban density and proportion of elderly residents. In several of the sensitivity analyses, these two variables showed significant odds between the municipalities included in the analysis and those excluded. Specifically, level of urban density showed a significant effect on ever and weekly smoking in 2021, for both models. The number of weekly smokers among the youth population is relatively low. Because a minimum youth population threshold was required for municipalities to be included in the dataset, this may have led to an overrepresentation of strongly urban municipalities, potentially masking existing effects.

Another explanation is that the differences in level of urbanization in the Netherlands – a densely populated country – are relatively small compared to other countries. Additionally, we had data available for a limited number of municipalities. The limited sample size may have reduced the statistical power, increasing the likelihood of Type II errors. As a result, some associations may not have reached statistical significance even if a true effect exists.

### Migration background

The only variable that significantly predicted ever smoking and weekly smoking in 2021 was the proportion of people with a migration background. In the Netherlands, about one-third of people with a migration background were born in Europe, and two-thirds outside Europe^24^. Most are of Turkish, Surinamese, or Moroccan origin. In these analyses, all groups were combined. Municipalities with a higher proportion of people with a migration background had a lower prevalence of youth smoking in 2021. Although migration background has been identified as a predictor of lower smoking rates in some studies^8^, most research shows that smoking prevalence is higher among immigrant populations than in the general population^25^. Culture, gender, and social differences between immigrants and the general population were considered as possible explanations. In both studies, all adult immigrant groups were grouped together as one predictor.

One possible explanation for finding a negative association between proportion of people with a migration background and smoking prevalence is that being surrounded by people with a migration background might have a protective effect for young people. One study found that having two immigrant parents and being a second-generation immigrant (i.e. born in the US) was associated with a protective effect against smoking^26^. One of the reasons suggested was the lower social acceptability of smoking among immigrant populations. Another study also found less tobacco use among immigrant youth and suggested that this could be explained by both family and individual factors^27^. Immigrant parents are less likely to use tobacco, and their children are less likely to mix with peers who smoke. Overall, in the Netherlands, we see that smoking is less popular among schoolchildren with a non-Dutch background. In the Netherlands, a national survey on smoking, vaping, and other substance use factors among schoolchildren showed that in 2023 fewer students between aged 12–16 years with a non-Dutch background had smoked at some point or in the past month compared with those with a Dutch background only^28^.

### Other relevant factors

Although our study variables failed to show a significant association with smoking rates, with the exception of migration level, we did see great variation in smoking prevalence rates among municipalities in 2021 and over time. The question therefore remains: how can these differences for 2021 and over time be explained? In every municipality in the Netherlands, the same national-level tobacco control regulations apply, including tobacco taxes, restrictions on availability of tobacco, indoor public smoking bans, a sales ban for individuals under 18 years, and plain packaging requirements^29^. We thus need to consider other factors.

One possible explanation for the differences between municipalities is the variation in how local interventions are chosen and implemented. A study among Dutch municipalities on local alcohol prevention policies showed that those municipalities with a greater reduction in alcohol consumption among youth populations tended to have implemented a broader range of alcohol control interventions. They combined educational strategies with regulatory measures, enforcement efforts, and media campaigns^30^. Even though many interventions in the Netherlands are not mandatory for municipalities, they can still play an important role in reducing smoking prevalence. One example of these approaches is ‘Growing up in a rewarding environment’ (Dutch: ‘Opgroeien in een kansrijke omgeving’). This intervention helps municipalities use local youth data to promote the health and wellbeing of young people and to prevent them from using alcohol, drugs and tobacco^31^. This approach is based on the Icelandic Prevention Model. Since adopting the model in the 1990s, Iceland has seen the largest drop in youth substance use in Europe^32^.

### Strength and limitations

Little research has been done on the association between characteristics of municipalities and local smoking rates in youth populations. To obtain the best possible understanding of which variables of municipalities are associated with smoking prevalence, we examined both policy-related and demographic factors. Unfortunately, complete data were only available for a limited number of municipalities. The sample sizes, and particularly for smoking over time, were quite small. Additionally, the variable ‘Smoke-Free Generation’ could not be included in the analysis due to too many missing values. We chose not to use imputation in our analyses as the sample size was too small for a reliable imputation. Also, inter-item correlation was generally low, further complicating imputation. In the case of the Location Monitor, the data indicate whether a municipality is actively engaged in the Smoke-free Generation Movement or not, which is a binary and policy-driven variable that cannot reliably be imputed without introducing substantial bias. The missing data may have prevented some factors from reaching significance in our models. We conducted an additional logistic regression analysis to account for this.

Unfortunately, the four policy-related variables had only been available for 2021. If they had been available for more years, it might have been interesting to compare several years of policy-related variables. As this study design is explorative, no causal inferences can be drawn from the associations observed. Despite this, this study is a first attempt to explain large differences in tobacco prevention between municipalities. As the role of municipalities becomes increasingly important for implementing tobacco control policies, other countries might also want to investigate characteristics of municipalities that affect this role. Future studies could look at trends over time or include other variables, such as school context variables.

## CONCLUSIONS

We investigated to what extent various municipalityspecific variables are associated with municipal youth smoking rates. Overall, we see that for almost all municipalities, smoking prevalence decreased between 2015 and 2021. Despite these substantial improvements, few of the examined factors showed a significant association. Only the proportion of residents with a migration background demonstrated a significant influence on ever and weekly smoking prevalence. Questions remain about which factors explain the variation in youth smoking rates between municipalities.

## Supplementary Material



## Data Availability

The data supporting this research cannot be made available due to reasons of sensitivity.
