# Supplementary material for: Investigating current and temporal variation in municipal youth smoking rates in the Netherlands: A multivariable regression analysis
Source: Tob Induc Dis. 2025 Oct 9;23:10.18332/tid/209127. doi: 10.18332/tid/209127 (PMC12510323; doi:10.18332/tid/209127)
Supplement: Supplementary file 1 [file TID-23-150-s1.pdf]

**Appendix A.**

**Figure 1. Distribution of differences in ever smoking rates (2021 minus 2015) in municipalities among the population aged 12-16 yrs in the Netherlands**

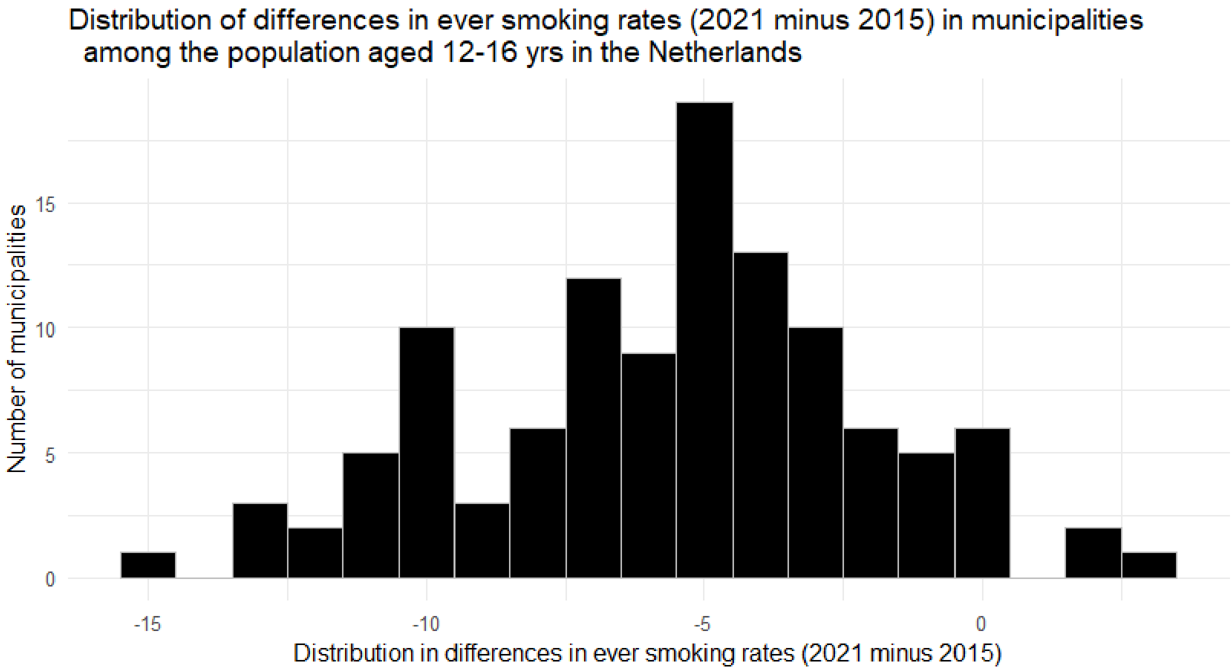


**Figure 2. Distribution of differences in ever smoking rates (2021 minus 2015) in municipalities among the population aged 12-16 yrs in the Netherlands**

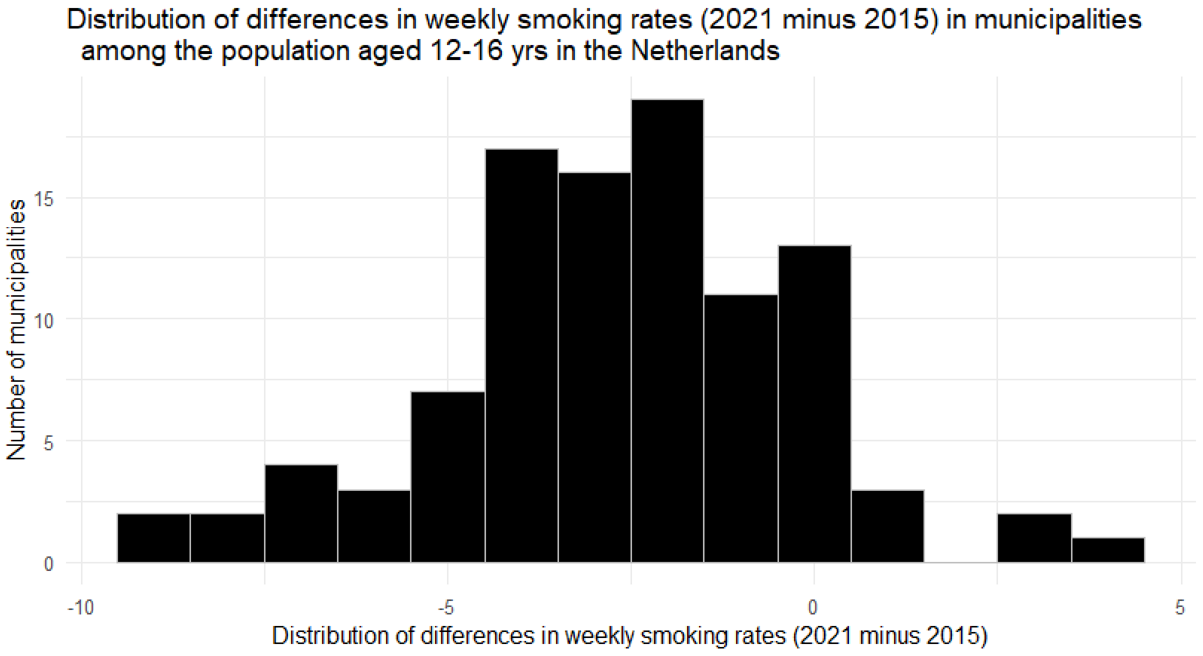

**Table S1.** *Multivariable regression analysis of the difference in ever smoking rates (2021 minus 2015) between 2015 and 2021 in municipalities among the population aged 12-16 yrs in municipalities in the Netherlands.*

| | Model 1[a] (N=113) | | | Model 2[b] (N=64) | | |
|---|---|---|---|---|---|---|
| **Variables** | **Beta** | **CI** | ***p*** | **Beta** | **CI** | ***p*** |
| Number of inhabitants per km² | 0.00 | -0.00 – 0.00 | 0.543 | 0.00 | -0.00 – 0.00 | 0.502 |
| Level of urban density | 0.28 | -0.90 – 1.45 | 0.641 | 0.29 | -1.17 – 1.74 | 0.70 |
| Mean standardized income | 0.05 | -0.18 – 0.28 | 0.661 | 0.01 | -0.24 – 0.26 | 0.94 |
| Proportion of residents with a migration background (%) | −0.03 | -0.21 – 0.14 | 0.691 | 0.02 | -0.20 – 0.24 | 0.861 |
| Proportion of elderly residents (%) | −0.00 | -0.26 – 0.25 | 0.986 | 0.2 | -0.14 – 0.46 | 0.293 |
| Proportion of adults with a low education (%) | 0.03 | -0.25 – 0.40 | 0.889 | 0.07 | -0.37 – 0.51 | 0.746 |
| Being actively involved in the Smoke-free Generation movement | - | - | - | NA | NA | NA |
| Smoking being incorporated in a local policy | - | - | - | -1.85 | -3.70 – 0.00 | **0.050** |
| Number of smoke-free location types | - | - | - | 0.01 | -0.39 – 0.41 | 0.961 |
| Tobacco control implementation grade | - | - | - | 0.32 | -0.52 – 1.16 | 0.453 |

[a] Model 1 includes the independent variables inhabitants per km2, level of urban density, standard income (mean), migration background, Proportion of elderly residents, and Proportion of adults with a low education (%).

[b] Model 2 includes the independent variables inhabitants per km2, level of urban density, standard income (mean), migration background, Proportion of elderly residents, Proportion of adults with a low education (%), Being actively involved in the Smoke-free Generation movement, Smoking being incorporated in a local policy, Number of smoke-free location types, and tobacco control implementation grade.

**Table S2.** *Multivariable regression analysis difference in weekly smoking difference (2021 minus 2015) between 2015 and 2021 in municipalities among the population aged 12-16 yrs in the Netherlands.*

| | **Model 1[a] (N= 100)** | | | **Model 2[b] (N=55)** | | |
|---|---|---|---|---|---|---|
| **Variables** | **Beta** | **CI** | ***p*** | **Beta** | **CI** | ***p*** |
| Number of inhabitants per km² | -0.00 | -0.00 – 0.00 | 0.922 | 0.00 | -0.00 – 0.00 | 0.814 |
| Level of urban density | −0.29 | -1.14 – 0.56 | 0.497 | -0.10 | -1.33 – 1.14 | 0.878 |
| Mean standardized income | −0.16 | -0.32 – 0.01 | 0.071 | -0.23 | -0.45 - -0.01 | **0.044** |
| Proportion of residents with a migration background (%) | 0.02 | -0.11 – 0.14 | 0.789 | 0.02 | -0.16 – 0.21 | 0.804 |
| Proportion of elderly residents (%) | 0.06 | -0.13 – 0.25 | 0.541 | 0.21 | -0.07 – 0.48 | 0.139 |
| Proportion of adults with a low education (%) | −0.20 | -0.48 – 0.07 | 0.145 | -0.20 | -0.60 – 0.20 | 0.329 |
| Being actively involved in the Smoke-free Generation movement | - | - | - | NA | NA | NA |
| Smoking being incorporated in a local policy | - | - | - | -1.09 | -2.81 – 0.62 | 0.206 |
| Number of smoke-free location types | - | - | - | -0.04 | -0.39 – 0.31 | 0.819 |
| Tobacco control implementation grade | - | - | - | 0.21 | -0.60 – 1.01 | 0.608 |

a Model 1 includes the independent variables inhabitants per km2, level of urban density, standard income (mean), migration background, proportion of elderly residents, and proportion of adults with a low education (%).
b Model 2 includes the independent variables inhabitants per km2, level of urban density, standard income (mean), migration background, proportion of elderly residents , proportion of adults with a low education (%), being actively involved in the Smoke-free Generation movement , smoking being incorporated in a local policy, number of smoke-free location types, and self-reported grade.

**Appendix B.**

**Table S3.** *Multivariable logistic regression between included and excluded municipalities of ever smoking in 2021 rates among people aged 12-16 in the Netherlands.*

| | Model 1[a] (N=264) | | | Model 2[b] (N=174) | | |
|---|---|---|---|---|---|---|
| **Variables** | **Log odds** | **Std. Error** | **p** | **Log odds** | **Std. Error** | **p** |
| Number of inhabitants per km² | -0.00 | 0.00 | 0.569 | 0.00 | 0.00 | 0.590 |
| Slightly urban | 0.30 | 0.43 | 0.482 | 0.04 | 0.73 | 0.953 |
| Moderately urban | 0.61 | 0.57 | 0.285 | 0.50 | 0.96 | 0.604 |
| Strongly urban | 3.04 | 1.15 | **0.008** | 3.87 | 1.96 | **0.048** |
| Very highly urban | 3.31 | 1.99 | 0.097 | 3.60 | 1.96 | 0.178 |
| Mean standardized income | 0.10 | 0.06 | 0.071 | 0.33 | 0.12 | **0.005** |
| Proportion of residents with a migration background (%) | -0.02 | 0.03 | 0.576 | -0.03 | 0.04 | 0.493 |
| Proportion of elderly residents (%) | -0.06 | 0.06 | 0.312 | 0.00 | 0.09 | 0.984 |
| Proportion of adults with a low education (%) | 0.18 | 0.08 | **0.030** | 0.26 | 0.12 | **0.033** |
| Being actively involved in the Smoke-free Generation movement | - | - | - | NA | NA | NA |
| Smoking being incorporated in a local policy | - | - | - | 0.09 | 0.56 | 0.871 |

| | | | | | | |
|---|---|---|---|---|---|---|
| Number of smoke-free location types | - | - | - | 0.20 | 0.11 | 0.077 |
| Tobacco control implementation grade | - | - | - | 0.28 | 0.24 | 0.250 |

a. Model 1 includes the independent variables inhabitants per km2, level of urban density, standard income (mean), migration background, Proportion of elderly residents , and proportion of adults with a low education (%). 301 municipalities were included. 48 municipalities were excluded.

b. Model 2 includes the independent variables inhabitants per km2, level of urban density, standard income (mean), migration background, proportion of elderly residents, proportion of adults with a low education (%), being actively involved in the Smoke-free Generation movement , smoking being incorporated in a local policy, number of smoke-free location types, and self-reported grade.

**Table S4.** Multivariable *logistic regression between included and excluded municipalities of weekly smoking in 2021 rates among people aged 12-16 in the Netherlands.*

| | Model 1[a] (N=264) | | | Model 2[b] (N=174) | | |
|---|---|---|---|---|---|---|
| Variables | **Log odds** | **Std. Error** | ***p*** | **Log odds** | **Std. error** | ***p*** |
| Number of inhabitants per km² | -0.00 | 0.00 | 0.880 | 0.00 | 0.00 | 0.955 |
| Slightly urban | 0.31 | 0.36 | 0.390 | -0.13 | 0.54 | 0.815 |
| Moderately urban | 0.94 | 0.49 | **0.055** | 0.73 | 0.73 | 0.318 |
| Strongly urban | 2.74 | 0.87 | **0.002** | 2.36 | 1.12 | **0.035** |
| Very highly urban | 3.26 | 1.70 | **0.055** | 2.94 | 1.95 | 0.132 |
| Mean standardized income | 0.05 | 0.04 | 0.234 | 0.13 | 0.07 | 0.067 |
| Proportion of residents with a migration background (%) | -0.02 | 0.03 | 0.526 | -0.03 | 0.03 | 0.320 |

| | | | | | | |
|---|---|---|---|---|---|---|
| Proportion of elderly residents (%) | -0.05 | 0.27 | 0.270 | -0.04 | 0.06 | 0.514 |
| Proportion of adults with a low education (%) | 0.09 | 0.18 | 0.177 | -0.04 | 0.09 | 0.176 |
| Being actively involved in the Smoke-free Generation movement | - | - | - | NA | NA | NA |
| Smoking being incorporated in a local policy | - | - | - | 0.13 | 0.42 | 0.757 |
| Number of smoke-free location types | - | - | - | 0.09 | 0.07 | 0.236 |
| Tobacco control implementation grade | - | - | - | 0.18 | 0.18 | 0.331 |

a. Model 1 includes the independent variables inhabitants per km2, level of urban density, standard income (mean), migration background, proportion of elderly residents , and proportion of adults with a low education (%).

b. Model 2 includes the independent variables inhabitants per km2, level of urban density, standard income (mean), migration background, proportion of elderly residents, proportion of adults with a low education (%), being actively involved in the Smoke-free Generation movement , smoking being incorporated in a local policy, number of smoke-free location types, and self-reported grade.

**Table S5**. *Multivariable logistic regression between included and excluded municipalities of difference ever smoking between 2015 and 2021 rates among people aged 12-16 in the Netherlands.*

| | Model 1[a] (N=113) | | | Model 2[b] (N=64) | | |
|---|---|---|---|---|---|---|
| Variables | **Log odds** | **Std. Error** | ***p*** | **Log odds** | **Std. Error** | ***p*** |
| Number of inhabitants per km² | 0.00 | 0.00 | 0.178 | 0.00 | 0.00 | 0.083 |
| Slightly urban | 0.47 | 0.39 | 0.229 | 0.58 | 0.58 | 0.313 |
| Moderately urban | 0.38 | 0.47 | 0.413 | 0.32 | 0.70 | 0.647 |

| | | | | | | |
|---|---|---|---|---|---|---|
| Strongly urban | 0.91 | 0.57 | 0.11 | 0.62 | 0.81 | 0.441 |
| Very highly urban | 0.13 | 1.03 | 0.902 | 0.21 | 1.34 | 0.878 |
| Mean standardized income | 0.01 | 0.03 | 0.798 | 0.03 | 0.04 | 0.459 |
| Proportion of residents with a migration background (%) | -0.00 | 0.02 | 0.851 | -0.00 | 0.03 | 0.923 |
| Proportion of elderly residents (%) | 0.10 | 0.04 | **0.020** | 0.17 | 0.06 | **0.004** |
| Proportion of adults with a low education (%) | 0.03 | 0.06 | 0.629 | 0.10 | 0.08 | 0.201 |
| Being actively involved in the Smoke-free Generation movement | - | - | - | NA | NA | NA |
| Smoking being incorporated in a local policy | - | - | - | -0.40 | 0.37 | 0.286 |
| Number of smoke-free location types | - | - | - | -0.02 | 0.06 | 0.781 |
| Tobacco control implementation grade | - | - | - | -0.26 | 0.15 | 0.087 |

a. Model 1 includes the independent variables inhabitants per km2, level of urban density, standard income (mean), migration background, proportion of elderly residents , and proportion of adults with a low education (%).

b. Model 2 includes the independent variables inhabitants per km2, level of urban density, standard income (mean), migration background, proportion of elderly residents , proportion of adults with a low education (%), being actively involved in the Smoke-free Generation movement, smoking being incorporated in a local policy, number of smoke-free location types, and self-reported grade.

**Table S6**. Multivariable *logistic regression between included and excluded municipalities of difference weekly smoking between 2015 and 2021 rates among people aged 12-16 in the Netherlands.*

| | Model 1[a] (N=00) | | | Model 2[b] (N=55) | | |
|---|---|---|---|---|---|---|
| Variables | **Log odds** | **Std. Error** | ***p*** | **Log odds** | **Std. Error** | ***p*** |
| Number of inhabitants per km² | 0.00 | 0.00 | 0.398 | 0.00 | 0.00 | 0.205 |
| Slightly urban | 0.40 | 0.42 | 0.332 | 0.07 | 0.59 | 0.911 |
| Moderately urban | 0.56 | 0.49 | 0.248 | 0.50 | 0.70 | 0.478 |
| Strongly urban | 1.27 | 0.59 | **0.031** | 0.83 | 0.81 | 0.307 |
| Very highly urban | 0.77 | 1.04 | 0.455 | 1.09 | 1.32 | 0.409 |
| Mean standardized income | 0.01 | 0.03 | 0.743 | 0.03 | 0.05 | 0.532 |
| Proportion of residents with a migration background (%) | -0.01 | 0.02 | 0.658 | -0.02 | 0.03 | 0.561 |
| Proportion of elderly residents (%) | 0.08 | 0.05 | 0.071 | 0.15 | 0.06 | **0.012** |
| Proportion of adults with a low education (%) | 0.06 | 0.06 | 0.289 | 0.14 | 0.08 | 0.096 |
| Being actively involved in the Smoke-free Generation movement | - | - | - | NA | NA | NA |
| Smoking being incorporated in a local policy | - | - | - | -0.70 | 0.39 | 0.069 |
| Number of smoke-free location types | - | - | - | -0.03 | 0.06 | 0.612 |

| | | | | | | |
|---|---|---|---|---|---|---|
| Tobacco control implementation grade | - | - | - | -0.07 | 0.16 | 0.651 |

a. Model 1 includes the independent variables inhabitants per km2, level of urban density, standard income (mean), migration background, proportion of elderly residents , and proportion of adults with a low education (%).

b. Model 2 includes the independent variables inhabitants per km2, level of urban density, standard income (mean), migration background, Proportion of elderly residents, proportion of adults with a low education (%), being actively involved in the Smoke-free Generation movement, smoking being incorporated in a local policy, number of smoke-free location types, and self-reported grade.

© 2025 Jooren S.J.A. et al.
